# Investigation of Magnetoelectric Properties and Applications in Multiferroic Composite

**DOI:** 10.3390/s26144418

**Published:** 2026-07-12

**Authors:** Tingyu Deng, Jinlou Gu, Dong Wang, Jie Jiao

**Affiliations:** 1 Key Laboratory for Ultrafine Materials of Ministry of Education, School of Materials Science and Engineering, East China University of Science and Technology, Shanghai 200237, China; 2 Shanghai Institute of Ceramics, Chinese Academy of Sciences, Shanghai 201800, China; jiejiao@mail.sic.ac.cn; 3 Center of Materials Science and Optoelectronics Engineering, University of Chinese Academy of Sciences, Beijing 100049, China; 4 University of Chinese Academy of Sciences, Beijing 100049, China

**Keywords:** magnetoelectric composites, resonator applications, wireless communication, magnetoelectric coupling, multiferroic materials

## Abstract

In this work, resonator applications based on the magnetoelectric coupling effect in multiferroic materials are systematically investigated, with particular emphasis on mechanically driven ME antennas. A finite-element model is established to analyze the electromechanical response and coupling behavior of the device. To better describe the converse ME process, a nonlinear magnetostrictive model is introduced to evaluate the influence of material properties, structural configuration, and DC bias magnetic field on resonance characteristics and radiation performance. The simulation results show that the radiation intensity of the ME antenna is strongly dependent on the applied bias magnetic field and can be significantly enhanced under the optimal operating condition. On this basis, key parameters are optimized to reduce the resonance frequency and improve the radiation response. Prototype devices are then fabricated and experimentally characterized. The measured results verify the predicted resonance behavior and demonstrate the feasibility of the proposed devices in low-frequency wireless communication and magnetic-anomaly sensing. This study provides theoretical guidance and experimental support for the design of portable low-frequency ME antenna systems and other resonator-based magnetoelectric devices.

## 1. Introduction

Magnetoelectric (ME) composites are functional material systems that enable energy conversion among magnetic, mechanical, and electric fields. Because of their multiphysics coupling characteristics and potential applications in weak magnetic-field detection, low-frequency communication, energy harvesting, and intelligent sensing, they have attracted increasing attention in recent years [1,2,3,4,5]. The ME effect describes the coupling between magnetic and electric fields. Under an alternating magnetic field, the magnetostrictive phase deforms and transfers strain to the piezoelectric phase, generating charge or voltage output, which is known as the direct ME effect. Conversely, under an alternating electric field, the piezoelectric phase vibrates through the converse piezoelectric effect [6,7,8,9,10], and the resulting strain is transferred to the magnetostrictive phase, inducing dynamic magnetization and radiating an oscillating magnetic signal, namely the converse ME effect [11,12,13]. These coupled effects provide the physical basis for ME resonators used in magnetic-field receivers and mechanically driven transmitting antennas.

ME materials have evolved from single-phase multiferroics to composite systems and further toward flexible, miniaturized, and integrated devices. Since single-phase materials usually exhibit weak room-temperature response and limited practical performance, composite structures combining magnetostrictive and piezoelectric phases have become the main research focus. Among different configurations, laminated ME composites are especially attractive because of their efficient interfacial strain transfer, simple fabrication, strong coupling response, low loss, and tunable structure. As a result, they have been widely investigated in weak magnetic-field sensors, ME-receiving devices, and low-frequency mechanically driven ME antennas [14,15,16].

For ME-receiving structures, previous studies mainly focused on improving conversion efficiency and reducing equivalent magnetic noise. Wang et al. [17] developed a “Multi-Push-Pull” ME-receiving antenna based on PMN-PT single-crystal fibers and interdigital electrodes, achieving an ME electric-field coefficient of 62 V/(cm·Oe), a charge coefficient of 2680 pC/Oe, and an equivalent magnetic noise of 5 pT/Hz^1/2^ at 1 Hz after low-noise amplification. Fang et al. [18] fabricated a Metglas/PIN-PMN-PT laminated ME composite in the longitudinal–longitudinal mode and obtained voltage coefficients of 17 V/Oe under quasi-static conditions and 147 V/Oe at resonance, with an equivalent magnetic noise of 8.6 pT/Hz^1/2^ at 1 Hz. Fang et al. [19] further proposed an in-plane series-connected MLT-type ME-receiving antenna based on PMN-PT fibers, which reduced capacitance and improved low-frequency magnetic-field detection sensitivity. These studies demonstrate the high sensitivity achievable in ME-receiving devices and provide useful support for integrated ME systems.

In comparison, mechanically driven ME antennas based on the converse ME effect have shown particular promise for low-frequency radiation and communication. In underwater, underground, and enclosed environments, conventional electromagnetic waves are strongly restricted by medium conductivity, propagation loss, and complex surroundings. Although very-low-frequency electromagnetic waves (VLF, 3–30 kHz) offer strong penetration capability, conventional VLF systems usually require extremely large facilities and high power consumption [20,21,22]. Unlike conventional antennas that radiate through oscillating currents, mechanically driven ME antennas generate radiation mainly through periodic oscillation of magnetic dipole moments under electromechanical resonance [23]. Their operating frequency is governed by acoustic or mechanical resonance rather than free-space wavelength, enabling low-frequency radiation with structures much smaller than the electromagnetic wavelength and partially relaxing the Chu-Harrington limitation for electrically small antennas [24].

Recent studies have verified the feasibility of this mechanism and promoted the development of compact low-frequency radiators. Dong et al. [25] confirmed that mechanically driven ME antennas follow the magnetic dipole radiation model and predicted the magnetic-field distribution from 1 m to 100 km. Niu et al. [26] demonstrated a miniaturized bulk PZT/Terfenol-D ME antenna, although fabrication complexity and interfacial reliability remain challenging. Chu et al. [27] reported a laminated ME antenna that achieved error-free communication over 18 m at 21.2 kHz with only 50 V_pp_ excitation. Deng et al. [28,29,30] further developed bending-mode ME antennas, reducing resonance frequency while maintaining relatively high radiation intensity. These results indicate that mechanically driven ME antennas provide an effective route toward miniaturized low-frequency communication systems.

Different from previous studies that mainly focused on the material properties, resonant responses, or optimization of a single magnetoelectric resonator structure, this study is oriented toward low-frequency near-field communication applications and systematically establishes the relationship among the structural dimensions, vibration modes, resonant frequencies, and magnetic-field output induced by converse magnetoelectric coupling in laminated magnetoelectric composite antennas. Finite-element simulations were performed to analyze the effects of dimensional parameters on the resonant characteristics and magnetic-flux-density generation capability of the ME antenna, and device fabrication and experimental measurements were further conducted to verify the resonant frequency, bias magnetic field response, and near-field magnetic output. On this basis, transmitting and receiving magnetoelectric antenna prototypes were designed and fabricated according to the requirements of the communication test, and near-field signal transmission was experimentally demonstrated. Therefore, the main contribution of this work lies in presenting a complete research route from structural parameter design, resonant-frequency tuning, and magnetic-field output optimization to communication-function verification, providing experimental evidence and design guidance for extending laminated magnetoelectric antennas from conventional resonator studies to low-frequency miniaturized communication applications.

## 2. Multiphysics Simulation of ME Composites

With the rapid development of multiphysics simulation software, the physical mechanisms involved in two-dimensional and three-dimensional simulations of magnetoelectric antennas can now be presented more clearly. In this work, the multiphysics coupling characteristics of a magnetoelectric antenna are investigated and discussed using the COMSOL finite element simulation platform [31,32,33,34].

According to the converse magnetoelectric multiphysics coupling process of the magnetoelectric antenna, its radiation intensity is closely related to the strength of the applied DC bias magnetic field during operation. When the optimal bias magnetic field is reached, the radiation intensity can increase by orders of magnitude. To characterize the properties of the piezoelectric and magnetostrictive materials, the constitutive equations of the piezoelectric material can be described by Equations (1) and (2). In this subsection, $S_{p}$ and $T_{p}$ denote the strain and stress of the piezoelectric layer, respectively; $D_{p}$ and E represent the electric displacement and electric field, respectively; and $d_{p}$ denotes the piezoelectric coefficient. $s^{E}$ denote the elastic compliance coefficient and $\varepsilon^{T}$ denote the dielectric permittivity.

(1) $$S_{p}={s^{E}T}_{p}+d_{p}E$$

(2) $$D_{p}=d_{p}T_{p}+\varepsilon^{T}E$$

Existing studies have mainly focused on the modeling and simulation of linear magnetostrictive materials, while discussions of nonlinear magnetostrictive behavior remain limited. Therefore, this work constructs a nonlinear magnetostrictive model to investigate the nonlinear material characteristics of the magnetoelectric antenna under the converse magnetoelectric effect. Using Equation (3), the strain of the magnetostrictive material under different magnetization conditions can be calculated from the magnetization intensity M, saturation magnetization intensity $M_{s}$ and the saturation magnetostriction constant $\lambda_{s}$. Equation (4) relates the strain $S_{m}$ to the stress $T_{m}$, where $C_{H}$ is the stiffness matrix, whose value is determined by the Young’s modulus $E_{s}$ and Poisson’s ratio γ of the material. Equation (5) defines the magnetization intensity of the material as a nonlinear curve jointly determined by the initial susceptibility χ, the effective magnetic field intensity, and the Langevin function given in Equation (6). In Equation (7), the piezomagnetic coefficient $d_{m}$ of the magnetic material is defined by the ratio of strain to the effective magnetic field $H_{eff}$.

(3) $$S_{m}=\frac{3}{2}\lambda_{s}(\frac{M}{M_{s}}{)}^{2}$$

(4) $$T_{m}=C_{H}[S_{0}-S_{m}(M)]$$

(5) $$M=M_{s}L(\frac{3\chi_{0}H_{eff}}{M_{s}})$$

(6) $$L=\coth(\frac{3\chi_{0}H_{eff}}{M_{s}})-\frac{M_{s}}{3\chi_{0}H_{eff}}$$

(7) $$d_{m}=\frac{\partial S_{m}}{\partial H_{\mathrm{eff}}}$$

Under the assumption of ideal coupling between the piezoelectric phase and the magnetostrictive phase, the corresponding boundary conditions can be derived.

(8) $$T_{1,p}t_{p}=-T_{3,m}t_{m}$$

(9) $$E_{3}=-\nabla V_{3}$$

The frequency-domain dynamic equilibrium equation of the magnetoelectric composite is given as follows:

(10) $$\rho \frac{\partial^{2}u}{\partial t^{2}}=\nabla T+F_{v}$$

(11) $$-\rho \omega^{2}u=\nabla T+F_{v}e^{i\phi }$$

The frequency-domain dynamic equilibrium equation of the magnetoelectric composite is given as follows, where rho denotes the material density, u is the displacement vector, T represents the stress, and $F_{v}$ denotes the total external force. Furthermore, Maxwell’s equations can be employed to calculate the specific values of the magnetic vector potential A, magnetic field H, magnetic flux density B, and other related parameters in the discretized mesh.

**Figure 1:**
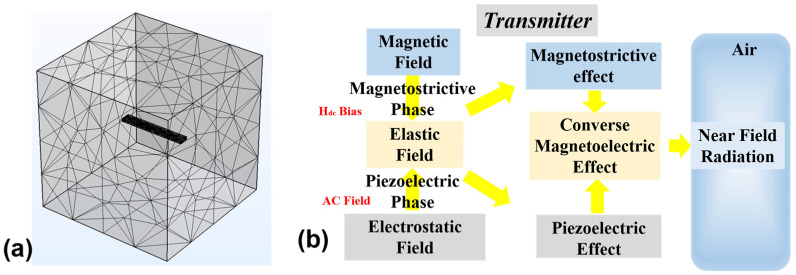
(**a**) Mesh generation of the three-dimensional structure of the magnetoelectric antenna; (**b**) diagram of the multi-physics field of converse magnetoelectric coupling.

Figure 1a illustrates the three-dimensional simulation model of the magnetoelectric antenna established using the finite element method. In the model, a sufficiently large cuboid represents the surrounding air domain, and the magnetoelectric antenna is located at the center and fully enclosed by the air region to emulate the realistic test environment in a dielectric medium. The entire geometry is discretized using an unstructured mesh. To ensure better convergence of the numerical results, a dense and uniform mesh is employed. The cuboid air domain has dimensions of 500 mm × 500 mm ×500 mm in width, so as to minimize the influence of boundary magnetic fields.

As shown in Figure 1b, the converse magnetoelectric coefficient of the laminated magnetoelectric composite was calculated in COMSOL 5.6 through coupling of the electrostatic, solid mechanical, and magnetic fields. First, a three-dimensional finite element model was established according to the actual laminated structure, and the material parameters, spatial arrangement, and interfacial coupling between the piezoelectric and magnetostrictive layers were defined. The interfaces were assumed to be perfectly bonded to ensure continuous displacement and stress transfer. Subsequently, the electrostatics, solid mechanics, and magnetic fields modules were configured and solved using multiphysics coupling. The applied potential difference generated an electric field in the piezoelectric layer and induced stress and strain through the converse piezoelectric effect. This mechanical response was then transferred to the magnetostrictive layer through the interface, leading to changes in its stress state and magnetization. Finally, based on the stress–magnetization coupling relationship of the magnetostrictive material, the magnetic fields module was used to calculate the magnetic field distribution and magnetic flux density variation inside and around the magnetostrictive layer, thereby characterizing the converse magnetoelectric response of the composite.

The previous subsection presented the theoretical description of the magnetoelectric response of the device and clarified the relationship between the main physical parameters and the output characteristics, thereby laying the foundation for the following analysis. Based on this, this subsection further investigates the key parameters under quasi-static operating conditions to reveal their influence on the magnetoelectric performance and application potential of the device. To facilitate comparison with existing mechanically driven magnetoelectric antennas, the laminated Metglas/PZT-5H/Metglas composite structure was adopted in the finite element simulation. The material properties of Metglas are listed in Table 1, while the piezoelectric layer is defined as PZT-5H using the built-in material parameters provided in COMSOL Multiphysics 5.6.

**Figure 2:**
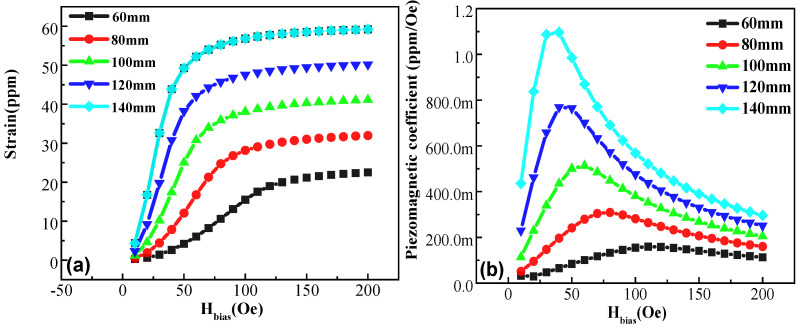
Performance variation with bias magnetic field at different spacings: (**a**) strain versus bias field; (**b**) piezomagnetic coefficient versus bias field.

Figure 2a shows the variation in the strain constant of the magnetostrictive material in the magnetoelectric antenna with the DC bias magnetic field under different length conditions. All curves exhibit a rapid increase in the low-bias-field region, indicating that the internal magnetic domains gradually rotate and that the magnetization process is enhanced. As the DC bias magnetic field further increases, the rate of strain increase gradually decreases and eventually approaches saturation, indicating that domain rotation is nearly complete and that the magnetostrictive effect is close to saturation. With increasing device length, the initial strain growth rate becomes faster, and the magnetic field required to reach the saturation strain decreases. This confirms that a longer device exhibits a more pronounced magnetostrictive response. Figure 2b presents the variation in the piezomagnetic coefficient with the DC bias magnetic field for devices with different lengths. The results show that the piezomagnetic coefficient first increases rapidly to a peak value and then gradually decreases with increasing DC bias magnetic field. At the peak point, the piezomagnetic effect is significantly enhanced, corresponding to the optimal converse magnetoelectric coefficient.

**Figure 3:**
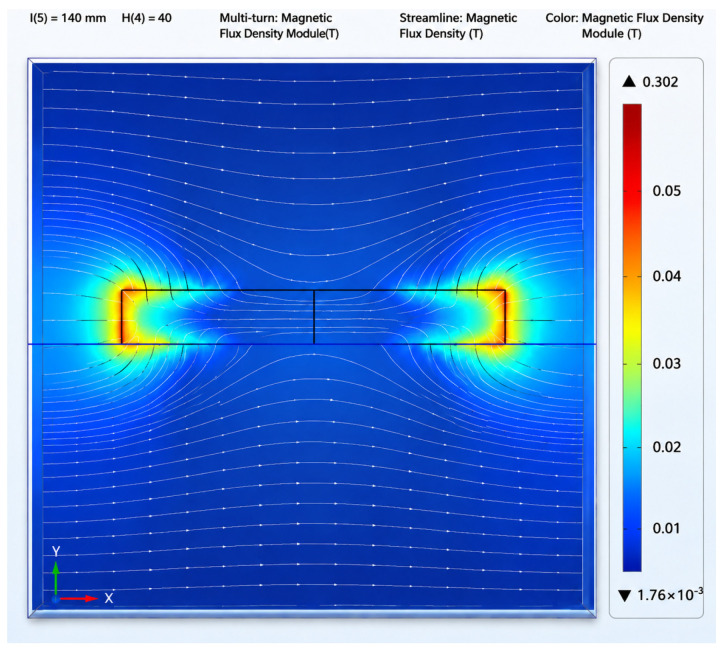
Contour map and streamline plot of the magnetic field distribution under an applied magnetic field 40 Oe.

Figure 3 presents the three-dimensional contour plot of the spatial magnetic flux density distribution of a ME antenna with a length of 140 mm under a DC bias magnetic field. It can be observed that the magnetic field becomes weaker with increasing distance from the device, while magnetic flux density accumulation appears near both ends of the magnetostrictive layer. The magnetic flux lines originate from one end of the magnetostrictive layer, pass through its interior, and return to the other end.

It should be noted that the magnetic flux density shown in composite is not an externally applied or controlled magnetic flux density, but rather the magnetic flux density generated in the magnetostrictive layer of the magnetoelectric antenna under AC voltage excitation through the converse magnetoelectric effect. The main purpose of this simulation is to investigate the influence of the structural dimensions of the magnetoelectric antenna on the magnetic-field generation capability inside the laminated magnetoelectric composite. To quantitatively compare the magnetic-field generation capability of devices with different dimensions, the average magnetic flux density within the volume region of the magnetostrictive layer on the same side of the magnetoelectric composite.

**Figure 4:**
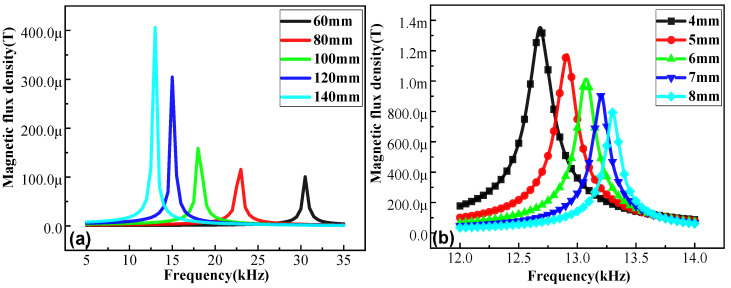
(**a**) Resonant frequency as a function of length; (**b**) resonant frequency as a function of thickness.

As shown in Figure 4a, the relationship between device length and resonance frequency is calculated under a fixed DC bias magnetic field of 6.25 Oe. It can be observed that magnetoelectric antennas of different lengths all exhibit distinct resonance peaks, and the magnetic flux density increases by multiple times at the resonance frequency. As the antenna length increases, the natural resonance frequency shifts and gradually decreases. This indicates that the CME coupling effect is strongest near the resonance point and gradually weakens as the operating frequency moves away from resonance. As shown in Figure 4b, the resonance frequency gradually increases with increasing thickness of the magnetic material. This is because a larger magnetic-layer thickness increases the overall Young’s modulus of the structure, thereby making the clamping effect on the piezoelectric phase more pronounced. These results indicate that adjusting the structural dimensions and material proportions of the ME antenna is crucial, as they significantly affect both the operating frequency and the radiation intensity of the device.

**Figure 5:**
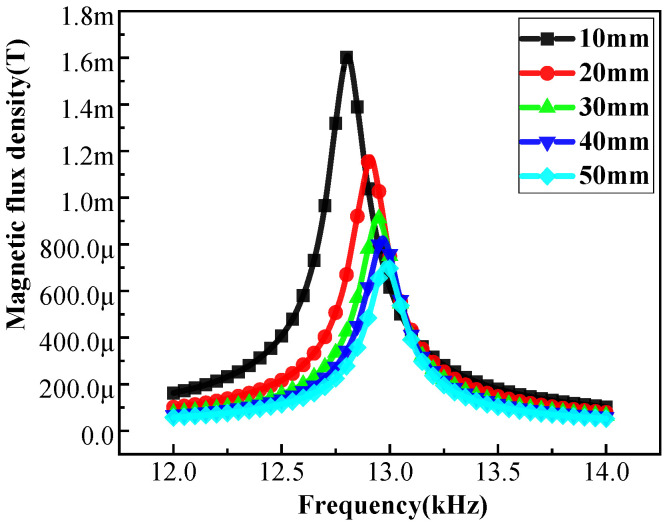
Resonant frequency as a function of width.

Figure 5 further shows the effect of device width on the resonance frequency of the magnetoelectric antenna under a fixed bias magnetic field of 6.25 Oe. The results indicate that the resonance frequency gradually increases as the width increases, although the growth rate progressively slows down. This is because the transverse demagnetization effect weakens with increasing width, while the stiffness effect gradually increases, leading to a higher resonance frequency. Meanwhile, the longitudinal extensional mode of the device gradually weakens, which causes the increase in resonance frequency to become less pronounced.

**Figure 6:**
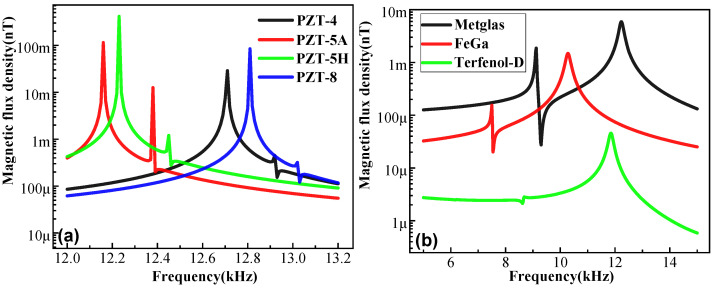
(**a**) Resonant frequency versus piezoelectric material; (**b**) resonant frequency versus magnetostrictive material.

Using the piezoelectric and magnetostrictive material parameters directly obtained from the built-in material library of COMSOL 5.6, Figure 6a compares the frequency-dependent radiation intensity of composites with different piezoelectric materials, namely PZT-4, PZT-5A, PZT-5H, and PZT-8, under identical excitation conditions and an identical volume of 160 mm × 20 mm × 2 mm. It can be seen that the ME antennas fabricated using PZT-5H and PZT-5A exhibit relatively higher radiation intensity across the entire frequency band and lower resonance frequencies. In contrast, the antennas fabricated using PZT-4 and PZT-8 show relatively weaker radiation intensity and higher operating frequencies. The simulation results indicate that the choice of piezoelectric material significantly affects the overall stiffness and damping characteristics of the ME antenna. In practical applications, the piezoelectric material should therefore be selected according to the target operating frequency, bandwidth, and application scenario.

Figure 6b illustrates the influence of magnetic materials on antenna performance, where three commonly used magnetostrictive materials, namely Metglas, FeGa, and Terfenol-D, are considered. The results show that, for the same volume, the ME antenna fabricated using Metglas exhibits the lowest operating frequency and the strongest converse ME coupling effect. This indicates that Metglas possesses the largest piezomagnetic coefficient and the most favorable magneto–mechanical coupling performance, enabling larger mechanical deformation near lower resonance frequencies. The antenna fabricated using FeGa exhibits an intermediate magnetic-field intensity between the other two materials. By contrast, the antenna based on Terfenol-D produces the lowest magnetic-field intensity because of its relatively small piezomagnetic coefficient. The simulation results demonstrate that the selection of the magnetostrictive-layer material is one of the key design parameters affecting the coupling response of ME antennas under both quasi-static and resonant conditions.

## 3. ME Coupling Performance Under Different Piezoelectric

In the previous subsection, the structural response and field distribution characteristics of the device were analyzed by finite-element simulation, which preliminarily revealed its ME coupling behavior and the key influencing factors, providing a basis for the subsequent performance study. On this basis, in order to further clarify the influence of piezoelectric-phase materials on the output characteristics of the device, this subsection focuses on the variation in ME coupling performance under different piezoelectric materials and discusses its significance for device optimization and practical applications based on the simulation results.

In this chapter, three Pb(Zr_x_Ti_1-x_)O_3_ (PZT) ceramics with different mechanical quality factors (Q) were customized as the piezoelectric phase materials. Their Q values were 50, 400, and 800, respectively. For convenience, they are hereafter denoted as PZT#1, PZT#2, and PZT#3. Each piezoelectric specimen had dimensions of 40 × 20 × 0.5 mm^3^. The ME antennas fabricated using these different piezoelectric materials were correspondingly denoted as Sample#1, Sample#2, and Sample#3. The parameters of different types of piezoelectric ceramics were listed in Table 2.

**Figure 7:**
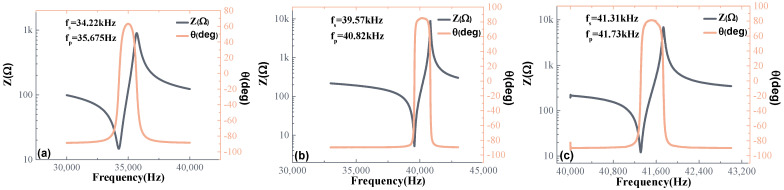
Impedance spectra of piezoelectric ceramics with mechanical quality factors of (**a**) 50, (**b**) 400, and (**c**) 800, respectively.

Figure 7 shows the impedance spectra of the three ME antennas. The frequency differences between resonance and antiresonance were 1.46 kHz, 1.24 kHz, and 0.42 kHz for PZT#1, PZT#2, and PZT#3, respectively. PZT#1 exhibited a resonance frequency of 34.22 kHz and an antiresonance frequency of 35.675 kHz, with relatively broad resonance features. PZT#2 showed a resonance frequency of 39.57 kHz and an antiresonance frequency of 40.82 kHz, with sharper impedance peaks and valleys. PZT#3 had a resonance frequency of 41.31 kHz and an antiresonance frequency of 41.73 kHz, exhibiting the sharpest resonance response among the three samples.

These results indicate that, with increasing mechanical quality factor, the resonance bandwidth gradually narrows, the resonance characteristics become more pronounced, and the frequency selectivity is significantly enhanced, demonstrating the typical narrowband response behavior of high-Q samples.

**Figure 8:**
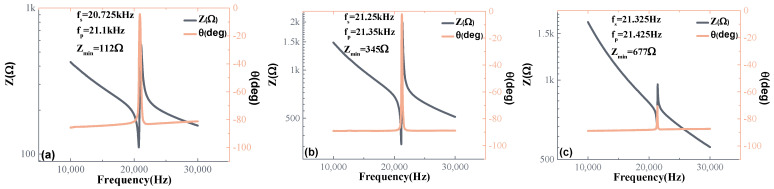
Impedance spectra of ME antennas (**a**) Sample#1, (**b**) Sample#2, and (**c**) Sample#3, respectively.

To construct an integrated transmitting-receiving ME antenna communication system, a laminated ME antenna with a length of 10 cm and a thickness of 4 mm was fabricated. The antenna consists of three Metglas layers and piezoelectric layers arranged in an M/P/M/P/M sandwich structure. Figure 8 shows the impedance spectra of the laminated ME antennas. All three samples exhibit typical series-resonance and parallel-antiresonance responses: a distinct impedance minimum |Z| appears near the series resonance frequency, followed by an antiresonance feature at a slightly higher frequency. This indicates significant electromechanical energy conversion within this frequency range and confirms the presence of a well-defined resonant operating point.

A comparison of the resonance parameters shows that the three samples exhibit similar resonance frequencies in the range of 20–22 kHz. Specifically, Sample#1 has a resonance frequency of 20.725 kHz, an antiresonance frequency of 21.1 kHz, and a minimum impedance (Z_min_) of about 112 Ω; Sample#2 shows 21.25 kHz, 21.35 kHz, and about 345 Ω; and Sample#3 shows 21.325 kHz, 21.425 kHz, and about 677 Ω, respectively. With increasing Q value, the resonance response becomes progressively narrower, indicating reduced equivalent damping, lower energy dissipation, and enhanced frequency selectivity.

**Figure 9:**
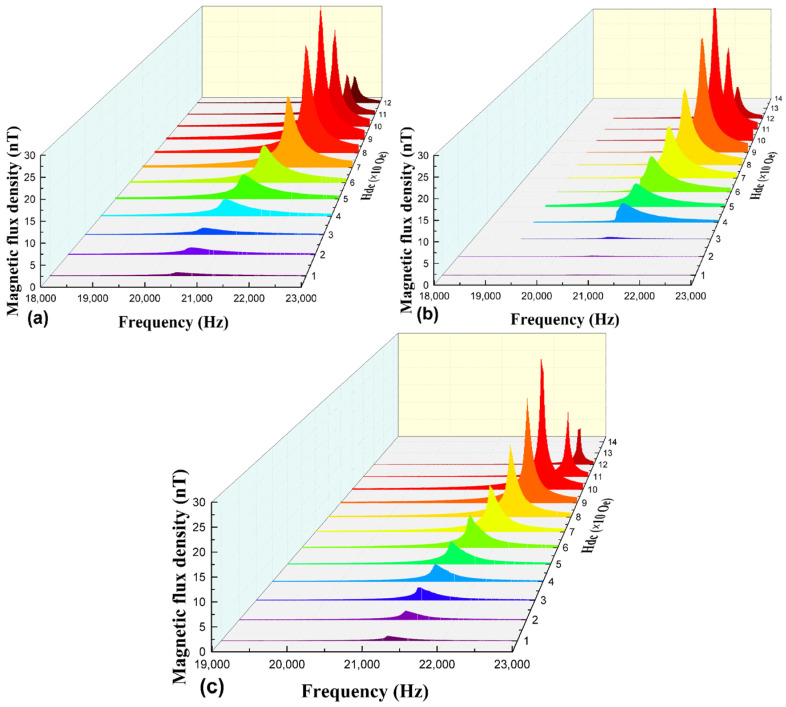
3D plots of magnetic flux density versus DC bias magnetic field for (**a**) Sample#1, (**b**) Sample#2, and (**c**) Sample#3.

The radiation performance of the three ME antennas was further compared experimentally. During the measurements, the receiving coil was fixed at 50 cm from the antenna center, and its relative position and orientation were kept unchanged. By gradually increasing the DC magnetic field, the three-dimensional magnetic flux density spectra as a function of frequency were obtained to evaluate the radiation response of the samples. As shown in Figure 9a, the radiation peak of Sample#1 increases with DC magnetic field and reaches about 41 nT at 90 Oe. Its relatively large full width at half maximum indicates a broad bandwidth, which is consistent with the response characteristic of a low-Q resonator. Figure 9b shows that the radiation peak of Sample#2 also increases with DC magnetic field and reaches about 36 nT at 100 Oe. Compared with Sample#1, the radiation peak is evidently narrower, indicating stronger resonance selectivity and a higher degree of energy concentration near resonance. As shown in Figure 9c, Sample#3 reaches a peak radiation intensity of about 33 nT at 100 Oe. Although its peak value is the lowest among the three samples, it exhibits the sharpest spectrum and the narrowest bandwidth, demonstrating the typical narrowband resonance characteristic of a high-Q device.

## 4. Applications of ME Composites in Wireless Signal Transmission

Based on the finite element simulation of the converse magnetoelectric effect in magnetoelectric composites, this subsection further investigates two magnetoelectric devices with different resonance frequencies, thereby providing a transition toward practical application verification. The reason for studying two devices with different resonance frequencies is that they correspond to different functional roles and application scenarios. The laminated Metglas/PZT-5H magnetoelectric antenna with a resonance frequency of approximately 4475 Hz is mainly designed for transmitting applications. On the one hand, this device is used to verify the reliability of the finite element simulation results and to further examine the influence of device volume on the near-field radiation capability. On the other hand, a magnetoelectric transmitting antenna with a lower resonance frequency is beneficial for exploring the feasibility of mechanically driven magnetoelectric antennas operating in the very-low-frequency and even lower-frequency bands. Since VLF/ULF electromagnetic signals generally exhibit relatively low attenuation in complex media, such as seawater, soil, and rock formations, designing magnetoelectric transmitting antennas with lower resonance frequencies and stronger radiation intensities through the volume effect may help mitigate the rapid attenuation of electromagnetic signals in such environments.

In contrast, the device with a resonance frequency of approximately 14.3 kHz corresponds to the magnetoelectric receiving element fabricated in this work. This device exhibits a relatively high magnetoelectric response and good magnetic-field detection capability near its resonance frequency, making it more suitable for use as a magnetic-field sensor or magnetic anomaly detection sensor. In other words, the 4.475 kHz device is mainly used to verify and explore the radiation performance of low-frequency magnetoelectric transmitting antennas, whereas the 14.3 kHz device is mainly employed to demonstrate the magnetic-field receiving and sensing capability of the magnetoelectric receiver.

Moreover, it should be noted that the resonance frequency of a magnetoelectric device is not a fixed value determined solely by the intrinsic properties of the constituent materials. Instead, it is strongly related to the device dimensions, laminated configuration, material parameters, boundary conditions, mechanical constraints, and electrical loading conditions. Therefore, it is reasonable and necessary to design magnetoelectric devices with different resonance frequencies according to different functional requirements.

**Figure 10:**
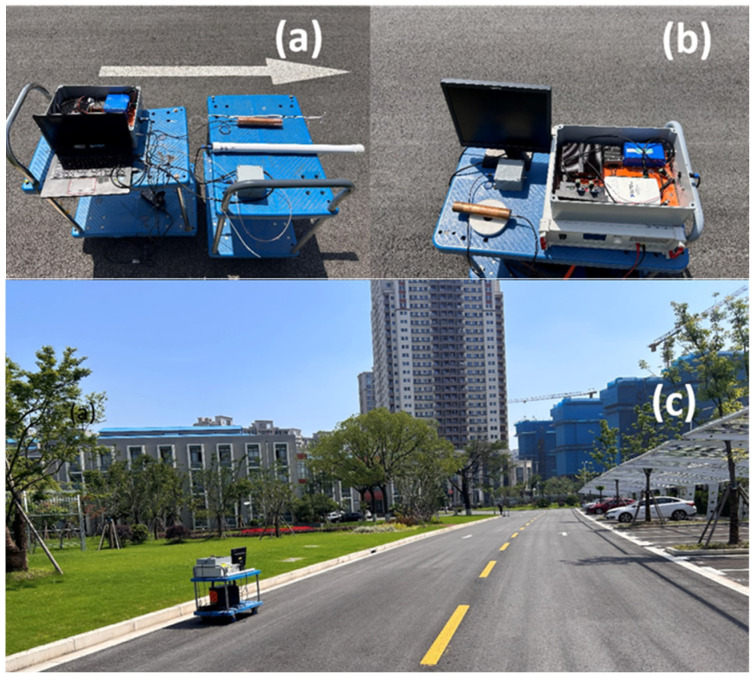
Integrated low-frequency magnetic-field communication and sensing system: (**a**) signal receiving end; (**b**) signal transmitting end; (**c**) long-distance communication system test scenario.

Figure 10a shows the architecture of the signal receiving unit in the proposed integrated low-frequency magnetic-field communication and sensing system, which mainly consists of a high-sensitivity magnetic detection receiving antenna, a ME antenna, a BNC connector, a portable power amplifier, a battery, and a signal acquisition module. Figure 10b presents the composition of the transmitting unit of the integrated system, including a high-power amplifier, a data acquisition card, a mobile power supply, and a modulated signal transmission module. Figure 10c shows a field-test photograph of the wireless communication distance measurement for the mechanical ME antenna, where the practical communication range was determined by adjusting the separation distance between the transmitter and receiver.

**Figure 11:**
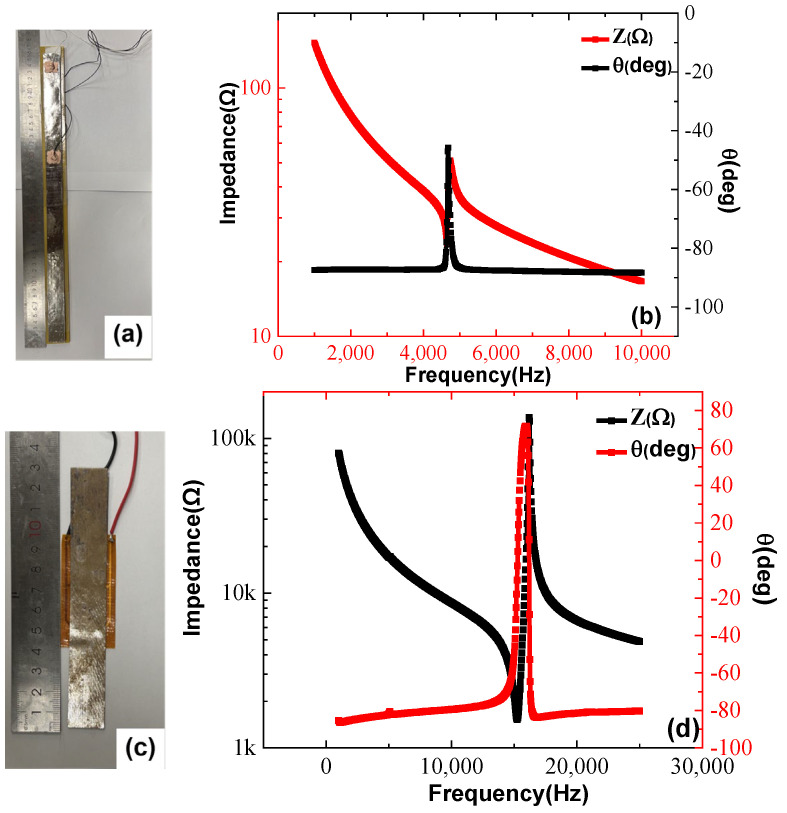
(**a**) Structural diagram and (**b**) impedance spectrogram of the transmitting end of the ME antenna; (**c**) Structural diagram and (**d**) impedance spectrogram of the receiving end of the ME antenna.

Figure 11a illustrates the structure of the designed laminated mechanical ME antenna. The antenna consists of three integrated Metglas laminates (400 mm × 20 mm × 0.025 cm, 36 layers in total) and two PZT ceramic layers placed on the upper and lower sides, respectively. The upper and lower ceramic plates are electrically connected in parallel. Figure 11b shows the impedance spectrum of the laminated ME antenna. The results indicate that the resonance frequency and antiresonance frequency are 4645 Hz and 4735 Hz, respectively. By tuning the antenna dimensions, the operating frequency can be significantly reduced, thereby providing a basis for communication applications in complex media, such as highly conductive environments, and effectively reducing propagation loss.

Figure 11c depicts the structure of the high-sensitivity ME-receiving antenna (140 mm × 20 mm × 0.025 cm, 4 layers in total). The core of the receiving antenna is a piezoelectric element composed of in-plane series-connected piezoelectric fibers, with two magnetostrictive strips attached to its upper and lower surfaces and bonded together using epoxy resin. The magnetostrictive material is magnetized along the longitudinal direction, while the piezoelectric material is polarized along the thickness direction. Finally, the electrical connection between the two phases is implemented using a flexible printed circuit board. Figure 11d presents the impedance characteristics of the magnetoelectric receiving antenna, whose resonance and antiresonance frequencies are 14.3 kHz and 16 kHz, respectively.

Previous studies have shown that the performance of ME composites strongly depends on the DC bias magnetic field, as the piezomagnetic coefficient of the magnetostrictive phase reaches a maximum near an optimal bias condition. To provide a compact and stable bias field, size-matched ferrite permanent magnets were attached to both ends of the mechanically driven ME antenna.

**Figure 12:**
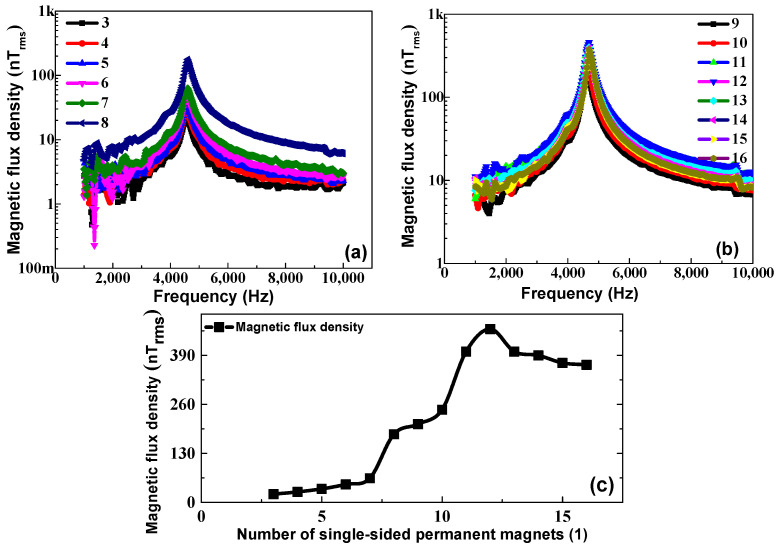
Relationship between radiation intensity and frequency of the ME composite under different numbers of bias magnets: (**a**) 3 to 8 bias magnets and (**b**) 9 to 16 bias magnets. (**c**) Relationship between the number of bias magnets and the radiation intensity measured at 1 m.

Figure 12a shows the frequency response of the antenna when the number of bias ferrites on each side increased from 3 to 8. The radiated magnetic-field intensity at 1 m increased significantly from about 20 nT to about 200 nT, indicating that an increase in bias magnetic field effectively enhances the radiation capability of the antenna.

Figure 12b presents the frequency response when the number of ferrites on each side was further increased from 9 to 16. The radiation intensity continued to increase and reached about 450 nT at 13 ferrites per side, but then gradually decreased with further increases in ferrite number, indicating the existence of an optimal bias magnetic field.

Figure 12c summarizes the relationship between ferrite number and radiation intensity at 1 m. The results show that the radiation intensity first increased rapidly, then reached a peak, and finally decreased, while the resonance frequency gradually shifted to higher values with increasing ferrite number.

These results confirm that the bias magnetic field is a key factor affecting both the radiation intensity and resonance characteristics of the mechanically driven ME antenna. The observed trend is consistent with the nonlinear magnetoelastic behavior of Metglas: the ME coupling is enhanced as the bias field approaches the optimal value, whereas excessive bias drives the material toward magnetic saturation and reduces the incremental magnetostriction. The resonance-frequency shift is likely related to the Delta-E effect, where the bias field changes the effective elastic modulus of the composite structure.

**Figure 13:**
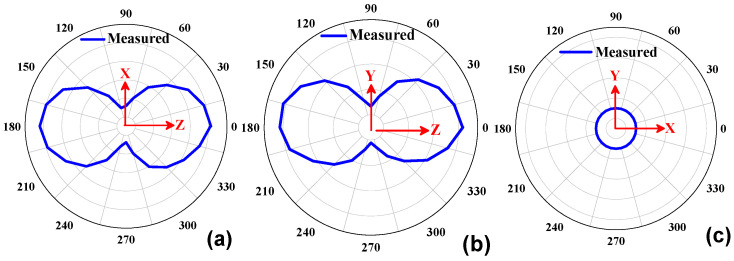
Spatial radiation patterns of the ME antenna in the (**a**) XOZ, (**b**) YOZ, and (**c**) XOY.

The radiation directivity of the mechanical ME antenna was further investigated. The measurements were carried out under a fixed radiation distance of 0.5 m, an applied voltage of 10 V_rms_, and an operating frequency of 4.45 kHz. The receiving coil was placed coplanar with and facing the transmitting antenna. The azimuth angle was defined as 0 deg when the antenna was parallel to the receiving coil, and the received signal was recorded every 15 deg during rotation. Figure 13a shows the variation in receiver voltage as a function of azimuth angle. The results indicate that the radiation intensity reaches its maximum at 0 deg and 180 deg, whereas it is minimized at 90 deg and 270 deg. Figure 13b exhibits a similar radiation directivity pattern. Further analysis reveals that no significant magnetic-dipole distribution is observed in the antenna cross-section, which is consistent with the weak radiation intensity monitored in Figure 13c. These results indicate that the mechanical ME antenna operates under voltage excitation, where the piezoelectric ceramic drives the magnetostrictive material to produce periodic magnetization oscillations, thereby radiating a magnetic field into space. This characteristic also confirms that the near-field radiation properties of the magnetoelectric antenna conform to the near-field distribution characteristics of a magnetic dipole. The magnetic and electric field components of a magnetic dipole can be expressed as follows, where $p_{m}$ denotes the magnetic dipole moment [35,36,37,38,39,40]. The specific formula is as follows:

(12) $$H_{r}=\frac{p_{m}\cos\theta }{2\pi \mu_{r}r^{3}}$$

(13) $$H_{\theta }=\frac{p_{m}\sin\theta }{4\pi \mu_{r}r^{3}}$$

**Figure 14:**
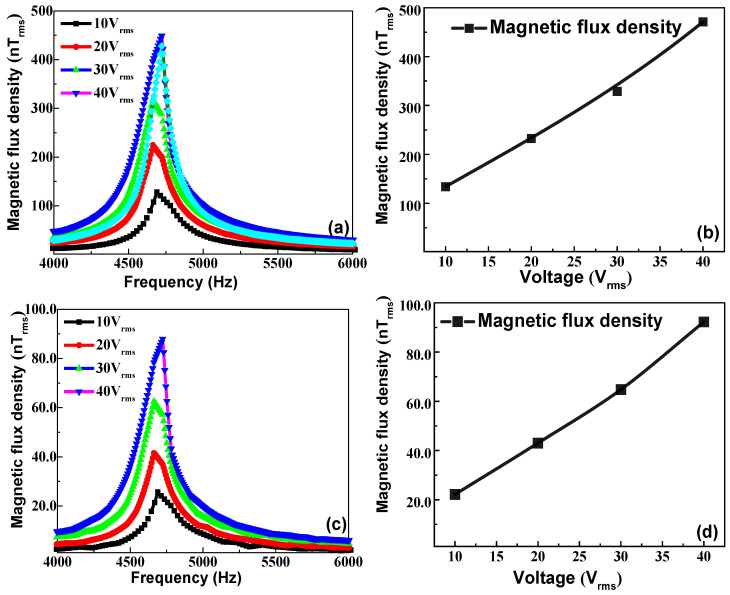
At a distance of 1 m: (**a**) frequency response curves of radiation intensity and voltage of the mechanically driven ME antenna; (**b**) relationship between radiation intensity and voltage. At a distance of 2 m: (**c**) frequency response curves of radiation intensity and voltage of the mechanically driven ME antenna; (**d**) relationship between radiation intensity and voltage.

The relationship between the radiation intensity and the excitation voltage of the ME antenna was further investigated, confirming an approximately positive correlation between the two. As shown in Figure 14a, at a distance of 1 m, the radiation intensity is approximately 130 nT under an excitation voltage of 10 V_rms_; when the voltage increases to 40 V_rms_, the radiation intensity increases to approximately 470 nT at 1 m. In addition, variations in the excitation voltage cause a shift in the antenna resonance frequency. This nonlinear behavior mainly originates from the Delta E effect of the magnetostrictive material and the electrostrictive behavior of the piezoelectric material. Figure 14b plots the radiation intensity as a function of voltage. Figure 14c shows the frequency–response curves of radiation intensity at a distance of 2 m under excitation voltages of 10 V_rms_ and 40 V_rms_. The results indicate that the radiation intensity is 23 nT at 10 V_rms_ and increases to 90 nT at 40 V_rms_. Figure 14d further illustrates the approximately linear relationship between radiation intensity and voltage. Based on the experimental results obtained under different distances and voltages, it is determined that the radiation intensity approximately follows a cubic decay law with distance. These results not only reveal the radiation characteristics of the mechanical ME antenna under different voltage excitations, but also provide an important basis for antenna performance optimization.

It should be emphasized that the simulated magnetic flux density and the measured magnetic flux density correspond to different physical regions. In the simulation, the magnetic flux density was obtained as the volume-averaged value within the magnetostrictive layer of the laminated magnetoelectric composite, which reflects the local magnetic-field generation capability of the device under the converse magnetoelectric effect. However, in the experiment, the magnetic flux density was measured in the external space at a certain distance from the magnetoelectric antenna. The external magnetic field of a compact magnetoelectric antenna can be approximated as the near field of a magnetic dipole, and the dominant magnetic field component decays approximately as $1/r^{3}$. Therefore, a significant reduction in the magnetic flux density is expected from the internal magnetostrictive layer to the external measurement point. Moreover, demagnetizing effects, mechanical and magnetic losses, interfacial bonding imperfections, fabrication tolerances, and measurement-system limitations further reduce the measured field. Consequently, the simulated internal magnetic flux density and the measured external magnetic flux density differ significantly in magnitude, while the simulation results are mainly used to reveal the relative trend of structural optimization.

**Figure 15:**
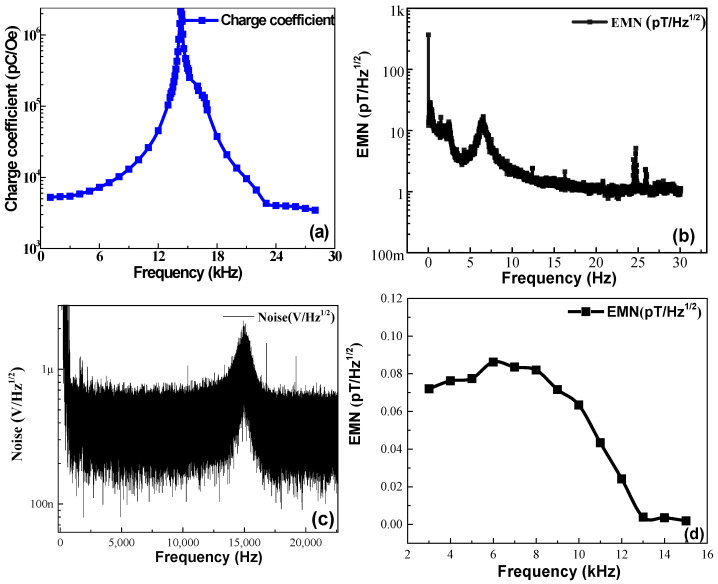
The ME receiver’s (**a**) direct ME effect and (**b**) detection sensitivity. (**c**) Noise of the receiving antenna; (**d**) equivalent magnetic field noise of the ME-receiving antenna.

To evaluate the application potential of the high-sensitivity ME-receiving antenna for low-frequency communication and magnetic-field sensing, its direct ME response and equivalent magnetic-field noise were characterized. Figure 15a shows the measured charge coefficient as a function of frequency. Under quasi-static operation at 1 kHz, the MLT-type receiving antenna exhibits a charge coefficient of 5200 pC/Oe. As the frequency approaches mechanical resonance, the charge coefficient increases sharply and reaches a maximum of 2.2 × 10^6^ pC/Oe, corresponding to an enhancement of about 434 times compared with the quasi-static state. This result confirms the strong resonance-enhanced ME coupling capability of the antenna.

Figure 15b shows the detection sensitivity of the receiving antenna after connection to a low-noise preamplifier. Under quasi-static conditions, the antenna achieves a sensitivity of 3.01 pT/Hz^1/2^ at 1 Hz, indicating excellent weak-field detection performance. This suggests that the in-plane series-connected MLT structure effectively reduces capacitance and noise, which is beneficial for low-frequency magnetic signal detection.

Figure 15c presents the noise spectrum of the receiving antenna from 1 kHz to 22 kHz, showing that the noise level varies across different frequency bands. Figure 15d further gives the equivalent magnetic-field noise, which is about 75 fT/Hz^1/2^ at 4475 Hz, near the operating frequency of the mechanically driven ME transmitting antenna. This noise floor is much lower than the transmitted magnetic-field strength over distances from several meters to tens of meters, indicating that the receiving antenna can provide sufficient signal-to-noise ratio for subsequent wireless communication experiments.

**Figure 16:**
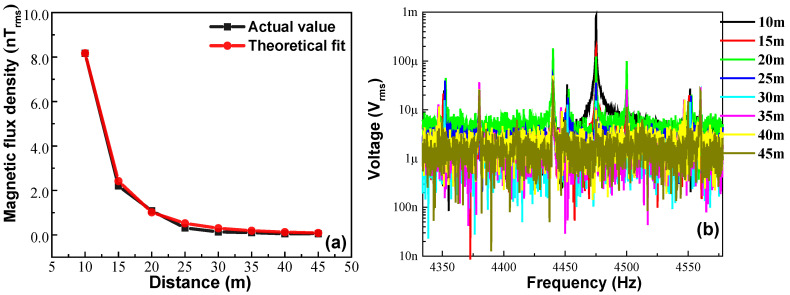
(**a**) The fitting of actual radiation distance with theoretical cubic decay; (**b**) the relationship between distance and radiation intensity in the spectrum diagram.

After completing the independent performance characterization of the transmitting and receiving antennas, the spatial propagation capability of the low-frequency magnetic-field communication system was further evaluated. In the experiment, the receiver position was fixed, and the position of the mechanically driven ME transmitting antenna was varied. The magnetic-field strength detected by the receiver at different distances was then measured, thereby obtaining the variation law of the radiated magnetic field with distance. The magnetic-field communication capability of the very-low-frequency ME antenna was further evaluated. During the experiment, the receiver position was fixed while the position of the ME transmitting antenna was varied to measure the relationship between magnetic-field strength and distance. Figure 16a shows that the mechanical ME antenna follows a magnetic-dipole radiation model, and its radiation intensity exhibits a cubic decay with distance, which is in good agreement with theory. The spectrum shown in Figure 16b indicates that environmental noise is the main factor limiting the wireless transmission distance, while it also allows the signal-to-noise ratio and transmission frequency to be directly observed.

**Figure 17:**
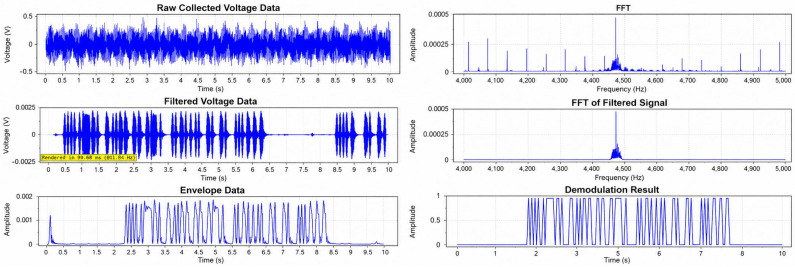
Using 2ASK to transmit the message “hi SICCAS” at a distance of 10 m to complete modulation and demodulation.

In Figure 17, we verify the practical information transmission capability of the proposed low-frequency magnetic-field communication system, a 10 m wireless communication experiment was conducted using 2ASK modulation based on a mechanically driven ME antenna. In the experiment, the carrier frequency was set to 4475 Hz, the transmission rate was 20 bps, and the excitation voltage was 40 V_rms_. The message “hi SICCAS” was encoded into a binary sequence and transmitted through the ME antenna.

**Figure 18:**
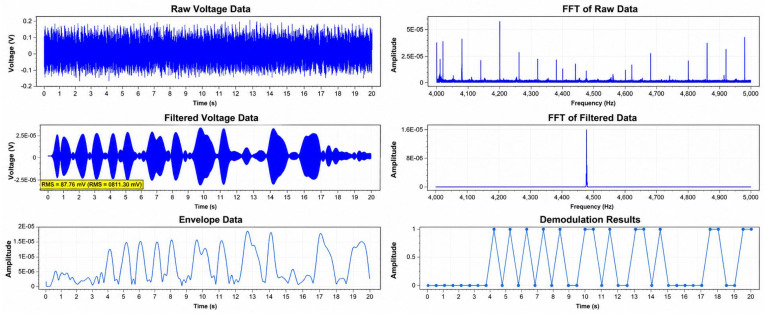
Using 2ASK to transmit the message “hi” at a distance of 30 m to complete modulation and demodulation.

To further evaluate the long-distance communication capability of the system, a 30 m wireless communication experiment was conducted using 2ASK modulation. In this test, the carrier frequency was set to 4475 Hz, the transmission rate was reduced to 2 bps, the excitation voltage was 40 V_rms_, and the transmitted message was “hi”. Figure 18 shows that the received signal still exhibited a clear spectral peak at 4475 Hz after FFT analysis, confirming that the carrier signal could be reliably detected at 30 m. After demodulation, the recovered bitstream was consistent with the original transmitted signal, and the message “hi” was successfully reconstructed.

These results demonstrate that the proposed system can still achieve reliable low-frequency wireless communication over a distance of 30 m, highlighting the potential of mechanically driven ME antennas for long-range low-frequency communication.

## 5. Conclusions

In this work, resonator applications based on the ME coupling effect in multiferroic materials were systematically investigated, with particular emphasis on a portable integrated low-frequency magnetic-field communication and sensing system. A multiphysics finite-element model was established in COMSOL to analyze the electromechanical coupling behavior, resonance characteristics, and radiation response of mechanically driven ME devices. By further introducing a nonlinear magnetostrictive model, the effects of material properties, structural parameters, and DC bias magnetic field on the converse ME process were clarified, providing theoretical guidance for device design and optimization.

Based on the simulation and optimization results, a stacked mechanically driven ME transmitting antenna based on a laminated Metglas/PZT structure was developed. Through structural dimension tuning, the resonance frequency was reduced to 4645 Hz. In addition, by employing an optimized ferrite-biased magnetic-field configuration with 13 magnet layers on each side, the radiated magnetic-field intensity reached 450 nT at 1 m and remained above 90 nT at 2 m, demonstrating an effective enhancement of low-frequency magnetic radiation. At the receiving end, an innovative MLT-type receiving antenna was adopted. Owing to its in-plane series-connected piezoelectric fiber structure, the equivalent capacitance was effectively reduced and the ME conversion capability was significantly improved. As a result, the charge coefficient increased from 5200 pC/Oe under quasi-static conditions to 2.2 × 10^6^ pC/Oe under resonant conditions, while the magnetic-field detection sensitivity reached 3.01 pT/Hz^1/2^ at 1 Hz and 75 fT/Hz^1/2^ at 4475 Hz.

System-level experiments further confirmed the feasibility of the proposed platform. Using 2ASK modulation at a carrier frequency of 4475 Hz, the system achieved error-free transmission of “hi SICCAS” at 20 bps over 10 m and reliable transmission of “hi” at 2 bps over 30 m. The measured radiation behavior was consistent with the magnetic dipole model, indicating that the field intensity decreases approximately with the cube of the transmission distance. Overall, this study demonstrates the feasibility of integrating low-frequency magnetic communication and magnetic sensing into a compact platform, and provides both theoretical and experimental support for future applications in ocean exploration, underwater monitoring, magnetic-anomaly sensing, and cross-medium communication in highly conductive environments.

## Figures and Tables

**Table 1:** Performance parameters of magnetostrictive Metglas.

Description	Symbol	Value	Unit
Conductivity	δ_M_	1.3 × 10^6^	S/m
Dielectric constant	ε_r_	1	1
Young modulus	E_s_	110	GPa
Poisson ratio	ν	0.37	1
Density of Metglas	ρ_m_	7180	kg/m^3^
Saturation magnetization	M_s_	1.89 × 10^4^	Oe
Saturation magnetostriction	λ_s_	30	ppm
Permeability of vacuum	μ_0_	4π × 10^−7^	H/m

**Table 2:** Summary of the parameters of the three piezoelectric materials.

	d_33_ (pC/N)	C_0_ (nF)	tanθ (‰)	f_r_ (kHz)	f_a_ (kHz)	d_31_ (pC/N)
PZT#1	520	41.51	13.6	38.6	39.8	215
PZT#2	330	19.42	19.4	39.7	41.0	147
PZT#3	170	14.36	14.3	40.1	40.8	63

## Data Availability

The original contributions presented in this study are included in the article. Further inquiries can be directed to the corresponding author.
